# Whole genome sequence analysis of multi-drug resistant and biofilm-forming *Staphylococcus haemolyticus* isolated from bovine milk

**DOI:** 10.1186/s12866-024-03575-z

**Published:** 2024-10-22

**Authors:** Daniel Jesuwenu Ajose, Tesleem Olatunde Abolarinwa, Bukola Opeyemi Oluwarinde, Peter Kotsoana Montso, Omolola Esther Fayemi, Adeyemi Oladapo Aremu, Collins Njie Ateba

**Affiliations:** 1 Antimicrobial Resistance and Phage Biocontrol Research Group (AREPHABREG), Department of Microbiology, School of Biological Sciences, Faculty of Natural and Agricultural Sciences, North-West University, Private Bag X2046, Mmabatho, 2735 South Africa; 2 Food Security and Safety Focus Area, Faculty of Natural and Agricultural Sciences, North-West University, Private Bag X2046, Mmabatho, 2735 South Africa; 3 Department of Microbiology, Faculty of Science, Stellenbosch University, Stellenbosch, 7600 South Africa; 4 Centre for Epidemic Response and Innovation, School for Data Science and Computational Thinking, Stellenbosch University, Stellenbosch, 7600 South Africa; 5 Department of Chemistry, Faculty of Natural and Agricultural Sciences, North-West University, Private Bag X2046, Mmabatho, 2735 South Africa; 6 Indigenous Knowledge Systems (IKS) Centre, Faculty of Natural and Agricultural Sciences, North-West University, Private Bag X2046, Mmabatho, 2735 South Africa

**Keywords:** Antibiotic resistance, Dairy, Food-pathogen, Milk, Food quality, Virulence

## Abstract

**Background:**

Milk is an excellent growth medium for microorganisms due to its nutritive composition. Microorganisms have been implicated in bovine mastitis (BM) in dairy cows as well as causing infections in animals and humans. Despite extensive endeavours to manage BM, this condition continues to persist as the most prevalent and economically burdensome problem affecting dairy cattle on a global scale. Non-aureus staphylococci (NAS) species such as *Staphylococcus haemolyticus*, *S. epidermidis*, and *S. xylosus* are currently the predominant microbiological agents identified as the main cause of subclinical udder infections and are also considered opportunistic pathogens in cases of clinical mastitis in dairy cows. Therefore, it is crucial to elucidate the genetic profile of these species. The primary objective of this study was to characterise three phenotypically determined multidrug-resistant NAS environmental strains (NWU MKU1, NWU MKU2, and NWU MKS3) obtained from dairy cows milk via whole-genome sequencing.

**Results:**

The results confirmed that the three isolates were *S. haemolyticus* with genome sizes of 2.44, 2.56, and 2.56 Mb and a G + C content of 32.8%. The genomes contained an array of antibiotic resistance genes that may potentially confer resistance to a range of antibiotic classes, such as macrolides, fluoroquinolones, aminoglycosides, cephalosporins, tetracyclines, peptides, and phenicol. Furthermore, all the genomes carried virulence genes, which are responsible for several functions, such as adhesion, enzyme and toxin production. The genomes of these organisms contained signatures encoding mobile genetic elements such as prophages and insertion sequences.

**Conclusion:**

These findings indicate there is a need for diligent monitoring with improved management practices and quality control strategies on farms to safeguard milk production systems and human health.

## Background

The most nutritive products of animal husbandry include milk and meat [1]. Milk is a nutrient-rich liquid produced by the mammary glands of mammals including cows [2]. It is an excellent source of proteins, vitamins, minerals, and nutrients for human consumption [3]. The composition of milk (amino acids high quality proteins, micronutrients, vitamins, and energy-containing fats) makes it an excellent growth medium for several microorganisms, hence very prone to microbial contamination [4–7]. The microbiological quality of milk is crucial in determining the quality and public health implications of its associated products. Moreover, the microbiological quality of milk at the point of milking from a healthy animal is theoretically expected to be safe for human consumption [8]. Although contamination of milk cannot be totally avoided, contamination can be significantly reduced by adhering to standard operating procedures and proper farm management techniques. Dairy products such as milk frequently contain a variety of bacteria contaminants, including *Escherichia coli*, *Klebsiella pneumoniae*, *Staphylococcus aureus*, *Streptococcus* species, and *Listeria* spp [1, 6, 9].

Studies have reported the presence of various multidrug resistant (MDR) pathogens in fresh and raw milk samples from dairy cows in the North West Province [10, 11] and Eastern Cape Province [12] of South Africa. MDR pathogens in food such as milk may result in staphylococcal food poisoning [13]. MDR infections result in higher morbidity and mortality rates, necessitate the use of costly medications, and increase the length of hospital stay [14]. Transmission of MDR pathogens from livestock (e.g. bovine) as well as through the consumption of contaminated food, necessitates the need for urgent control strategies [15]. Adherence to strategies aimed at limiting the transmission of typical MDR foodborne pathogens such as *E. coli*, *Shigella*, *Campylobacter* species, *Salmonella* Typhi, and nontyphoidal salmonellae, which have been advocated for by the Centers for Disease Control and Prevention is imperative [16]. However, the potential of unspecified agents with insufficient data to estimate agent-specific burdens to contaminate the food chain and cause complications to consumers highlights the need for continuous surveillance to help improve food quality and protect human health. Decreased levels of pathogen sensitivity and proper hygiene measures could aid in preventing the onset of disease, despite the use of contemporary strategies such as proper milking techniques [17, 18].

Non-aureus staphylococci (NAS) constitute a diverse collection of bacteria and have been consistently found to be the most often detected bacteria in milk samples, with studies reporting the NAS colonisation of the teat apex in both lactating and dry cows [19–21]. Additionally, NAS colonisation has been detected in the teat canal [22] and other parts of the udder [23]. In a recent study, *S. haemolyticus*, along with other species, such as *S*. *hominis*, *S*. *kloosii*, *S*. *rostri*, and *S*. *xylosus*, was detected in rectal faeces [24]. This finding suggested that cows can transmit NAS to the farm environment, which may contaminate the udder and thus lead to the development of intramammary infection (IMI). It is hypothesised that this infection occurs following colonisation of the teat apices. Among the several *Staphylococcus* species, *S. aureus* is widely recognised as the most pathogenic strain. The *S. haemolyticus* group is considered to be the most prominent etiological agent responsible for intramammary infections (IMIs) in dairy cattle, mostly involving colonisation of the teat surface. While certain staphylococcal species exhibit lower levels of virulence than *S. aureus*, they are nonetheless capable of colonising humans and potentially contributing to opportunistic infections [25, 26].

*Staphylococcus haemolyticus* has been recognised as a remarkably adaptable opportunistic species [27]. This strain is commonly found in human blood cultures [25] and ranks second to *S. epidermidis* in terms of frequency of isolation. *S. haemolyticus* is a commensal skin microbiota and is frequently detected in the axillary, perineal, and inguinal regions of individuals [28]. It also has the potential to induce septicaemia, peritonitis, otitis, urinary tract infections, and bovine mastitis [29]. Furthermore, the strain is widely recognised for its ability to develop resistance to several drugs and has a long-standing reputation for quickly acquiring resistance to methicillin and glycopeptide antibiotics [30, 31]. Its resistance to multiple antibiotics coupled with its ability to form biofilms contribute to the pathogenicity and virulence of this strain. However, the challenges and limitations encountered in the identification of *S. haemolyticus* by biochemical techniques have been documented [32].

Whole-genome sequencing (WGS) represents a contemporary technique used for the investigation of resistance mechanisms, especially in various pathogenic organisms. This technology has the capacity to process many DNA sequences simultaneously with high throughput at a reduced cost and time, hence providing extensive information on the genes contained within a pathogen genome [33, 34]. In addition, the technique has demonstrated considerable efficacy, leading to a paradigm shift in the field of microbial genome research. Furthermore, WGS has been used for the examination of a wide range of genetic disorders and mutation in various pathogens such as *S. haemolyticus*. Therefore, this study evaluated three environmental strains of *S. haemolyticus* isolated from milk samples with the aim of assessing their MDR, virulence and biofilm-forming characteristics and elucidating the genetic factors that contribute to their propensity for frequent phenotypic variations.

To fulfil the global increase in demand for milk driven by an increase in population in Asia, Latin America and Africa [35], particularly in South Africa [11], the food-animal production sector is expanding quickly. Most dairy farms in the Mafikeng suburb (North West Province, South Africa) are small, independently owned operations that typically provide fresh milk to both processing facilities and the surrounding community for on-site consumption. With such practices, milk meant for human consumption may become contaminated and ultimately pose public health risks to consumers, mainly resulting from improper hygiene practices during the milking process. The need for continuous surveillance of milk and other animal products for the presence of MDR bacterial foodborne pathogens is eminent. The data generated will contribute to both the Global Action Plan to tackle AMR and the South African antimicrobial resistance (AMR) national strategy framework (https://knowledgehub.health.gov.za/system/files/elibdownloads/2020-03/AMR%20National%20Action%20Plan%202018%20-%202024.pdf). The aim of this study was to characterise MDR and biofilm-forming BM pathogens from milk collected from apparently healthy cows (with not clinical signs of disease) and establish their public health significance.

## Methods

### Study design, study site, and ethical consideration

A randomised study design was used to perform a bacteriological analysis of raw and tank milk samples from the Molelwane farm in the Northwest Province, South Africa.

This study was conducted at the North‒West University Agricultural Research Farm (coordinates 25°47’30.4"S 25°37’22.1"E), Molelwane village, Mafikeng town, North West Province, South Africa. The study site was chosen because it also supplies milk and poultry products to the inhabitants of neighbouring communities in the area.

Ethical clearance for the study was obtained from the Faculty of Natural and Agricultural Sciences Research Ethics Committee (FNASREC) at North‒West University (NWU), and the ethical clearance number NWU-01311-22-A9 was assigned to the study.

### Sample collection

Sixty milk samples comprising aseptically individual raw cow’s milk (*n* = 40) as described by Goddens et al. [36] and bulk tank milk (*n* = 20) were randomly sampled over a two-week period.Each 250 mL milk sample was collected, correctly labelled, and transported on ice to the laboratory for microbial analysis. All the samples were analysed within 4 h of collection. All the microbiological culture materials used were obtained from Merck (Pty) Ltd (South Africa).

### Isolation and preliminary characterization of pathogens

Pathogens were isolated from the samples using the procedures outlined by Bissong and Ateba [11] with minor modifications. Briefly, aliquot of 10 µL for each sample was spread-plated on mannitol salt agar (MSA). All the plates were incubated at 37 °C for 24 h. Following the morphological characteristics exhibited by the colonies, the pure colonies were kept in tryptic soy broth (TSB) supplemented with 40% (v/v) glycerol at -80 °C for subsequent analysis.

### Phenotypic antibiotic susceptibility testing

The antimicrobial resistance profiles of all the isolates were determined against a panel of 8 antibiotics, namely, tetracycline (30 µg), linezolid (30 µg), gentamicin (10 µg), clarithromycin (15 µg), sulfamethoxazole-trimethoprim (25 µg), ciprofloxacin (5 µg), chloramphenicol (30 µg), and amoxicillin (10 µg), using the Kirby–Bauer disk diffusion technique [37]. Aliquots (100 µL) of bacterial suspension (comparable with 0.5 McFarland’s standards) prepared with 0.8% (w/v) sterile saline were spread-plated on Mueller Hinton agar (Merck, South Africa). The plates were incubated aerobically at 37 °C for 18–24 h. After incubation, the inhibition zones were measured in millimeters, and the results were interpreted based on recommended standards of the Clinical Laboratory Standards Institute [38], to classify the isolates as sensitive, intermediate resistant or resistant to a particular antibiotic. The multi-antibiotic resistance (MAR) phenotype and index were also determined following methods described by Krumperman [39].

### Determination of biofilm formation

#### Phenotypic detection of biofilm

The crystal violet-based microtiter plate test technique described by Mahmoodi [40] was used to assess the ability of the MDR isolates to form biofilms. Loop-rich cultures were inoculated and grown overnight in tryptic soy broth (TSB) at 37 °C. An aliquot of 10 µL of each overnight culture (10^5^ CFU/mL) was diluted in 190 µL of TSB. The 96-well microtiter plate was then filled with 200 µL of the diluted culture mixture and incubated at 4 °C, 25 °C, and 37 °C for 24, 48, and 72 h each. *S. aureus* ATCC 25,923, was used as a positive control while negative control well contained only 200 µL of TSB. The experiment was run in triplicates. After incubation, the TSB broth was discarded by flipping the plate upside down and then shaking off the broth containing the free cells. Phosphate-buffered saline (PBS) was then used to rinse the plates. To remove unattached cells, the washing procedure was repeated twice. The wells were stained with 200 µL (1% w/v) of crystal violet dye. The plates were left at room temperature for 1 h. Subsequently, the dye was washed off using PBS. This process was repeated five times to ensure that the dye was properly washed. Using blotting paper, the microtiter plate was air-dried at room temperature. After that, each well received 200 µL of 95% ethanol, the plate were left at room temperature for 5 min. An automatic enzyme-linked immunosorbent assay (ELISA) microtiter plate reader (MB-580, Zhengzhou, China) was used to measure the optical density (OD) of the resultant solution at a wavelength of 630 nm. According to Papa et al. [41], the following conditions were employed to study the isolates’ capacity to produce biofilms: ODS < ODC = no biofilm formation, ODC < ODS < 2ODC = weak biofilm formation, 2ODC < ODS < 4ODC = moderate biofilm formation, 4ODC < ODS = strong biofilm formation; where ODC = OD of negative control, ODS = OD of sample. To determine the OD for each isolate, ODs were measured in triplicate, and the mean value was used.

#### Detection of biofilm coding genes

The intercellular adhesion (*ica*) operon, encoded by the icaADBC gene, is responsible for the production of slime. Hence, the isolates were screened for the presence of *ica-* A and D genes. The polymerase chain reaction (PCR) protocols used were described by Omidi et al., and Atshan et al. [42, 43] respectively.

### Molecular characterisation and identification of isolates

#### Extraction of genomic deoxyribonucleic acid (gDNA) from bacterial colonies

Genomic deoxyribonucleic acid (gDNA) was extracted using the Zymo Research Genomic DNA™ Tissue MiniPrep Kit (Zymo Research, Irvine, CA, USA) in accordance with the manufacturer’s instructions. The purity of the extracted DNA was assessed using a Nanodrop™ Lite spectrophotometer (Thermo Scientific, Walton, MA, USA). DNA samples with A260/280 ratios ranging from 1.80 to 2.00 were classified as high purity DNA.

#### Preliminary molecular identification of isolates

The isolates were identified through the amplification of 16 S rRNA (341-F: AGAGTTTGATCCTGGCTCAG; 907-R: AAGGAGGTGATCCAGCCGCA) [44] and staphylococcal *gap* gene (GF-1: ATGGTTTTGGTAGAATTGGTCGTTTA; GF-2: GACATTTCGTTATCATACCAAGCTG) [45] specific regions.

### Whole genome sequencing - quality control, trimming, assembling and annotation

Three isolates (NWU MKU1, NWU MKU2, and NWU MKS3) that exhibit resistance to multiple drugs were subjected to whole-genome sequencing (WGS) analysis. The DNA samples were sent to Agricultural Research Council (ARC), Pretoria for whole genome sequencing. Briefly, the libraries were generated using the MGI Universal DNA library prep kit following the manufacturer’s instructions. The MGI DNBSEQ-G400 platform was utilised to perform paired-end sequencing for a total of 312 cycles. The quality assessment of the raw sequenced reads was conducted using the online program KnowledgeBase (KBase) v2.7.11 (https://www.kbase.us). FastQC v0.12.1 [46] was used for this purpose. Subsequently, the reads were filtered to remove low-quality reads and adapter regions using Trimmomatic v0.36 [47]. The de novo genome assembly was performed using SPAdes v3.15.3 [48], followed by annotation using the Prokka v1.14.5 annotation pipeline [49] and the Pathosystems Resource Integration Centre (PATRIC) annotation tool v.3.31.12 (https://www.bv-brc.org) [50]. A circular genome map of the isolates was constructed by uploading the annotated genome into the Proksee online database (https://www.proksee.ca) [51]. Moreover, a subsystem analysis of each genome was performed using the Comprehensive Genome Analysis (CGA) tool in the PATRIC online database. Furthermore, genome sequences with annotations were submitted to the National Centre for Biotechnology Information (NCBI) GenBank, where they were assigned accession numbers.

#### Determination of resistome, virulome and mobile genetic elements (MGEs)

The functional annotation data obtained from the Prokka and PATRIC annotation processes were utilised to search for a variety of antibiotic resistance genes and virulence determinants that hold therapeutic significance. The detection of resistance genes was performed using Resistance Gene Identifier (RGI) v6.0.2, which is a tool available on the Comprehensive Antibiotic Resistance Database (CARD) v3.2.7 platform (https://card.mcmaster.ca/analyze/rgi) [52]. The virulence factor database (VFDB) version 4.0 (http://www.mgc.ac.cn/cgi-bin/VFs/v5/main.cgi) [53] was utilised to determine the pathogenic determinants for each genome. Furthermore, the identification of mobile genomic elements (MGEs) was accomplished using the Proksee online database, accessible at (https://www.proksee.ca) [51]. The evaluation of MGE in the genome of the isolates was conducted by uploading the contigs of each isolate to the MGEfinder v1.0.3 database, which is hosted on the web platform of the Centre for Genomic Epidemiology (https://cge.food.dtu.dk/services/MobileElementFinder/) [54] and the Phage Search Tool Enhanced Release (PHASTER) web server (https://phaster.ca) [55].

#### Phylogenetic analysis

Phylogenetic analysis of the strains NWU MKU1, NWU MKU2, and NWU MKS3 involved comparing their evolutionary relationships with a set of high-quality genomes of *Staphylococcus* strains. This analysis was performed using the PATRIC Bacterial Genome Tree platform [50]. Specifically, the Comprehensive Genome Analysis report incorporates the reference and representative genomes provided by PATRIC into its phylogenetic analysis. Identification of the most closely related and representative genomes was accomplished using the Mash/MinHash algorithm. The phylogenetic location of the PATRIC global protein families (PGFams) was determined by selecting them from the genomes under consideration [56]. The protein sequences derived from these families were aligned using the MUSCLE algorithm, after which the corresponding nucleotide sequences for each protein were mapped to the protein alignment [57]. The amino acid and nucleotide alignments were combined into a single data matrix, which was subsequently subjected to analysis using RaxML [58]. Fast bootstrap was employed to create the support values in the resulting tree [59].

## Results

### Isolation and preliminary characterization of pathogens

With respect to the morphological and biochemical characteristics, a total of 72 presumptive staphylococcal isolates were recovered from 60 milk samples. Out of the 72 isolates, 48 (66.7%) were coagulase positive, while the remainder were coagulase negative (24 [33.3%]).

### Phenotypic antibiotic susceptibility

The coagulase-negative staphylococci (CONS) were subjected to antimicrobial susceptibility tests against 8 antibiotics, each representing a different class of antibiotics. The results revealed that only one-fifth (5 [20.8%]) of the CONS isolates were MDR, while 12 (50.0%) were resistant to fewer than 3 different classes of antibiotics, and 7 (29.2%) were susceptible to all antibiotics tested. The isolate was considered MDR if it showed resistant to at least three or more antibiotics belonging to different groups. MDR phenotypes were observed against ciprofloxacin, amoxicillin, trimethoprim/sulfamethoxazole, tetracycline and linezolid. The multi-antibiotic resistance phenotypes and indices are shown in Table 1.

**Table 1:** Multi-antibiotic resistant (MAR) phenotypes and MAR index of isolates

Resistant phenotype	Frequency	MAR Index
Cip-Amo-Tet-Cla-Lin-Gen-Chl- Tri/Sul Amo-Tet-Cla-Lin-Gen-Chl- Tri/Sul Amo-Cla-Lin	5 4 4	1.00 0.88 0.38
Cip-Amo- Tri/Sul	3	0.38
Amo-Tet-Lin	2	0.38
Cip-Gen	2	0.25
Amo-Lin	2	0.25
Amo	1	0.13
Tri/Sul	1	0.13

### Biofilm formation

**Fig. 1:**
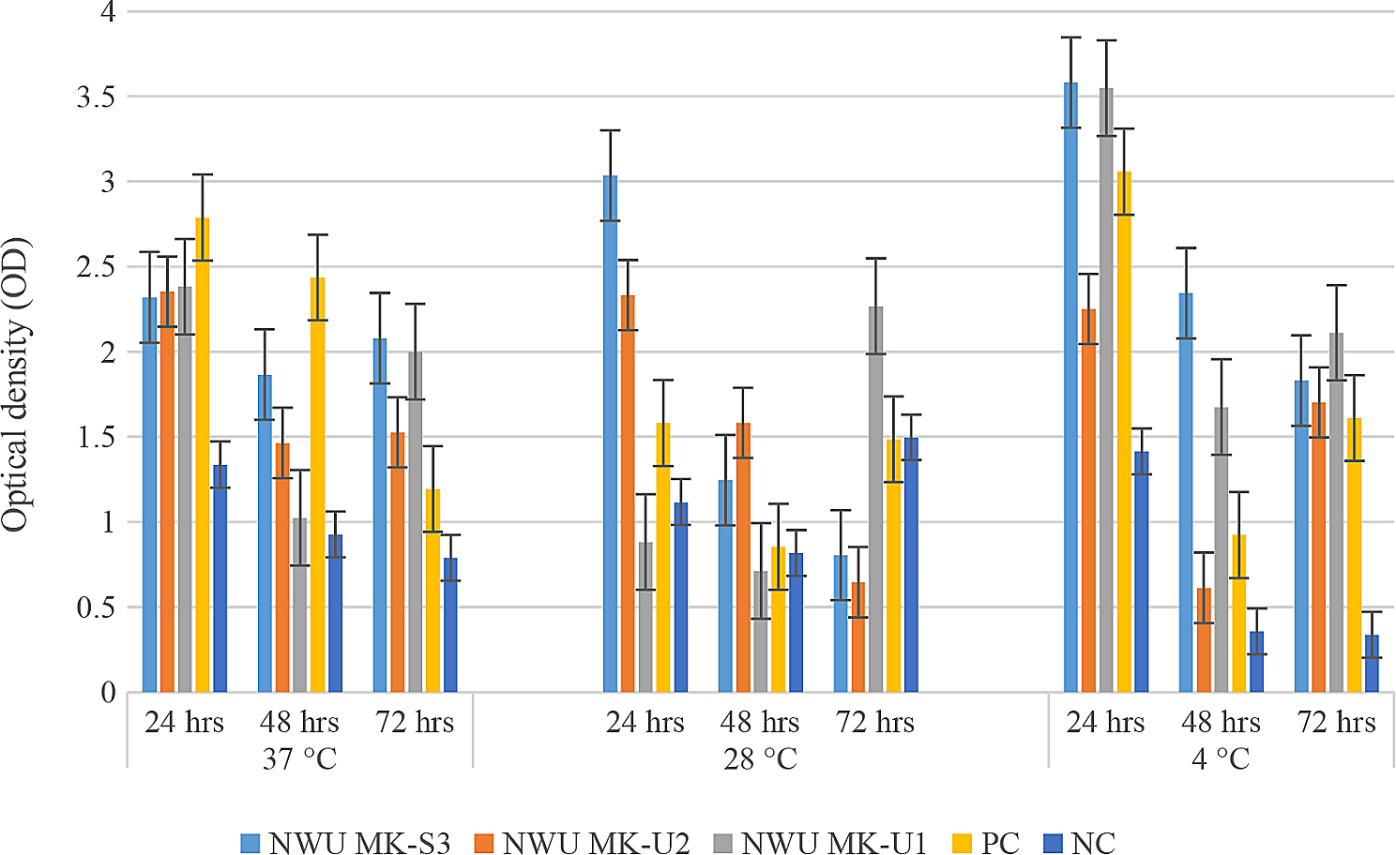
Phenotypic biofilm formation by *Staphylococcus* isolates at different temperature levels and incubation times. PC – positive control, NC – negative control

The isolates showed various biofilm formation patterns at different temperatures and time periods (Fig. 1). The results ranged from no biofilm production to strong biofilm production. While the biofilm formation capacity was directly proportional to the incubation period at 4 °C, it was inversely proportional to the incubation period at 28 °C. Nonetheless, at 37 °C, all the isolates showed weakly adherent after 24 h of incubation. NWU MK-U2 isolate revealed similar results across all incubation time periods. While the ability of the isolate NWU MK-S3 to form biofilm improved with increasing incubation time (moderate at 48 and 72 h), isolate NWU MK-U1 showed weak biofilm formation at 48 h of incubation but improved to produce moderate biofilm formation at 72 h. PCR results showed that no isolate showed amplification of the *ica* A or *ica* D genes.

### Preliminary molecular identification of isolates

**Fig. 2:**
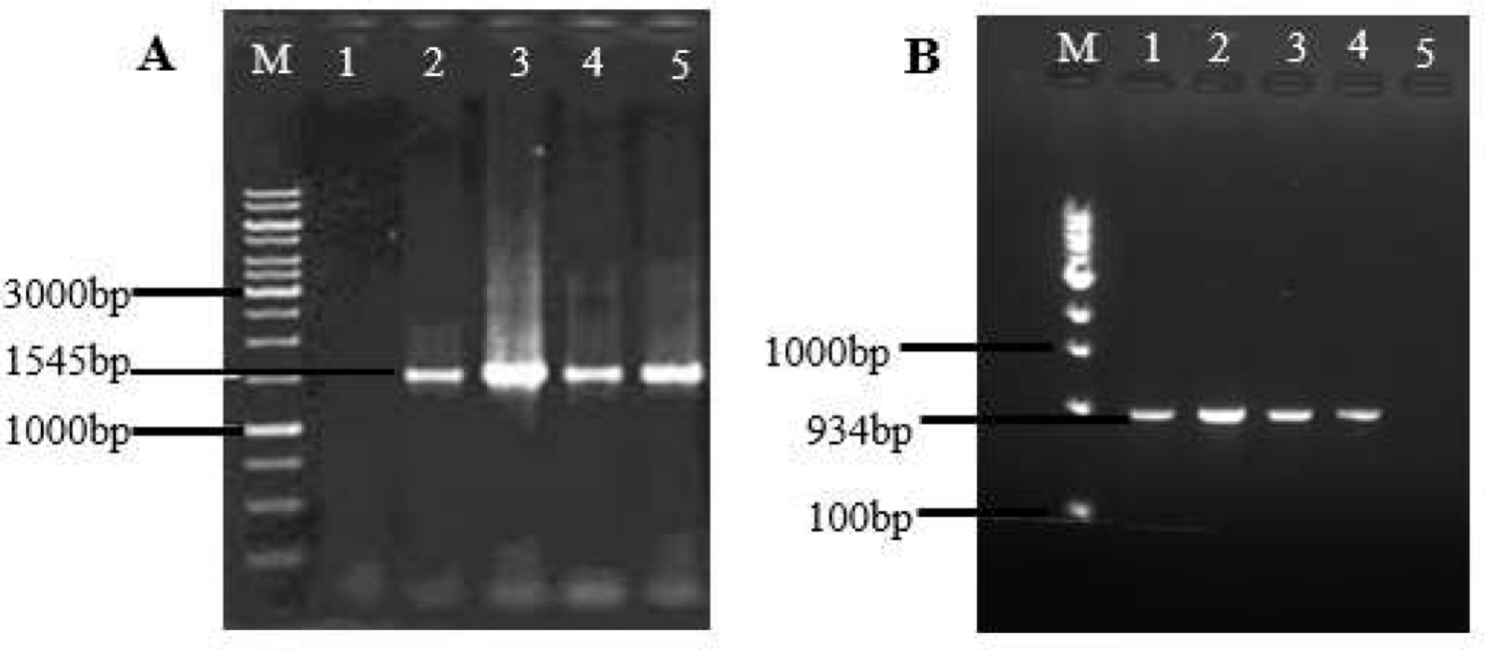
Identification of isolates by preliminary molecular techniques. (**A**) = 16 S rRNA gene, M – DNA marker (1 kb), lane 1: negative control (molecular grade water), lane 2: positive control (S. aureus ATCC 25923), lanes 3–5: samples; (**B**) = gap gene, M – DNA marker (100 bp), lane 1: positive control (S. aureus ATCC 25923), lanes 2–4: samples; lane 5: negative control (molecular grade water)

As shown in Fig. 2, the validation of the isolates’ *Staphylococcus* genus membership was achieved through the amplification of the 16 S region and the *gap* gene.

### Whole genome sequence analysis

After annotation and comparative evaluation against other genomes of the same species that were accessible through public databases, including NCBI and PATRIC, it was confirmed that the isolates’ genomes were of high quality. The subsequent data are presented, including the characteristics of the genomes, G + C content and genome length; genes of interest, including virulence and antibiotic resistance genes (ARGs); functional classification via subsystems; and a phylogenetic tree constructed through phylogenetic analysis.

#### The characteristics of the genome assembly

The genome assembly sizes of the NWU MKU1, NWU MKU2, and NWU MKS3 isolates were 2,444,508, 2,567,762, and 2,567,728 base pairs (bp), with G + C content of 32.85, 32.83, and 32.83%, and 21, 26, and 26 contigs, respectively. The genomes contained 2409, 2585, and 2485 protein-coding sequences (CDS), and 59, 61, and 61 noncoding RNAs, which included tRNAs and rRNAs, respectively. The N50 values for the genomes were 294,618 bp for NWU MKU1 and 304,465 bp for both NWU MKU2 and NWU MKS3, with an L50 value of 3 for each genome (Table 2).

**Table 2:** Assembly characteristics and taxonomy of the annotated genomes

Assembled genome content	Isolate		
	NWU MKU1	NWU MKU2	NWU MKS3
Coarse consistency (%)	99.9	99.9	99.9
Fine consistency (%)	99.9	99.7	99.7
Completeness (%)	100	100	100
Genome length (bp)	2,444,508	2,567,762	2,567,728
Contigs N50 (bp)	294,618	304,465	304,465
GC content (%)	32.85	32.83	32.83
Contigs	21	26	26
Contigs L50	3	3	3
Domain	Bacteria	Bacteria	Bacteria
Phylum	Firmicutes	Firmicutes	Firmicutes
Class	Bacilli	Bacilli	Bacilli
Order	Staphylococcales	Staphylococcales	Staphylococcales
Family	Staphylococcaceae	Staphylococcaceae	Staphylococcaceae
Genus	*Staphylococcus*	*Staphylococcus*	*Staphylococcus*
Species	*Staphylococcus haemolyticus*	*Staphylococcus haemolyticus*	*Staphylococcus haemolyticus*
Sequence read archive (SRA)	SRR26105960	SRR26105954	SRR26105961
Accession number	JAVSMG000000000	JAVSMH000000000	JAVSMI000000000
Number of CDS	2,409	2,585	2,485
Number of tRNA	56	58	58
Number of Repeat Regions	0	0	0
Number of rRNA	3	3	3
Number of hypothetical proteins	415	508	507
Number of proteins with functional assignments	1,994	2,077	2,077
Number of proteins with EC number assignments	728	727	727
Number of proteins with GO assignments	604	600	600
Number of proteins with Pathway assignments	542	540	540
Number of proteins with PATRIC genus-specific family (PLfam) assignments	2,380	2,547	2,546
Number of proteins with PATRIC cross-genus family (PGfam) assignments	2,397	2,564	2,563

The genome annotation encompassed both putative proteins and proteins with established functional assignments. The proteins included in the functional assignments consisted of proteins with Enzyme Commission (EC) numbers, gene Ontology (GO) assignments, and proteins that were mapped to Kyoto Encyclopedia of Genes and Genomes (KEGG) pathways. The PATRIC annotation encompasses two distinct categories of protein families, namely, genus-specific protein families (PLFams) and cross-genus protein families (PGFams). These protein families are found within the genomes under consideration.

The genomes of the three test isolates were identified as belonging to the genus *Staphylococcus* and the species *haemolyticus*. Therefore, the isolates were considered as *S. haemolyticus* NWU MKU1, *S. haemolyticus* NWU MKU2 and *S. haemolyticus* NWU MKS3. In particular, the assembled genome sequences of the test strains NWU MKU1, NWU MKU2 and NWU MKS3 in the NCBI GenBank were assigned the following accession numbers: JAVSMG000000000, JAVSMH000000000 and JAVSMI000000000, respectively. The genomes of the isolates subjected to annotation were assigned distinct genome identifiers, specifically 1279.3809, 1279.3808, and 1279.3813. The assembly and annotation details of the genomes are presented in Table 2.

**Fig. 3:**
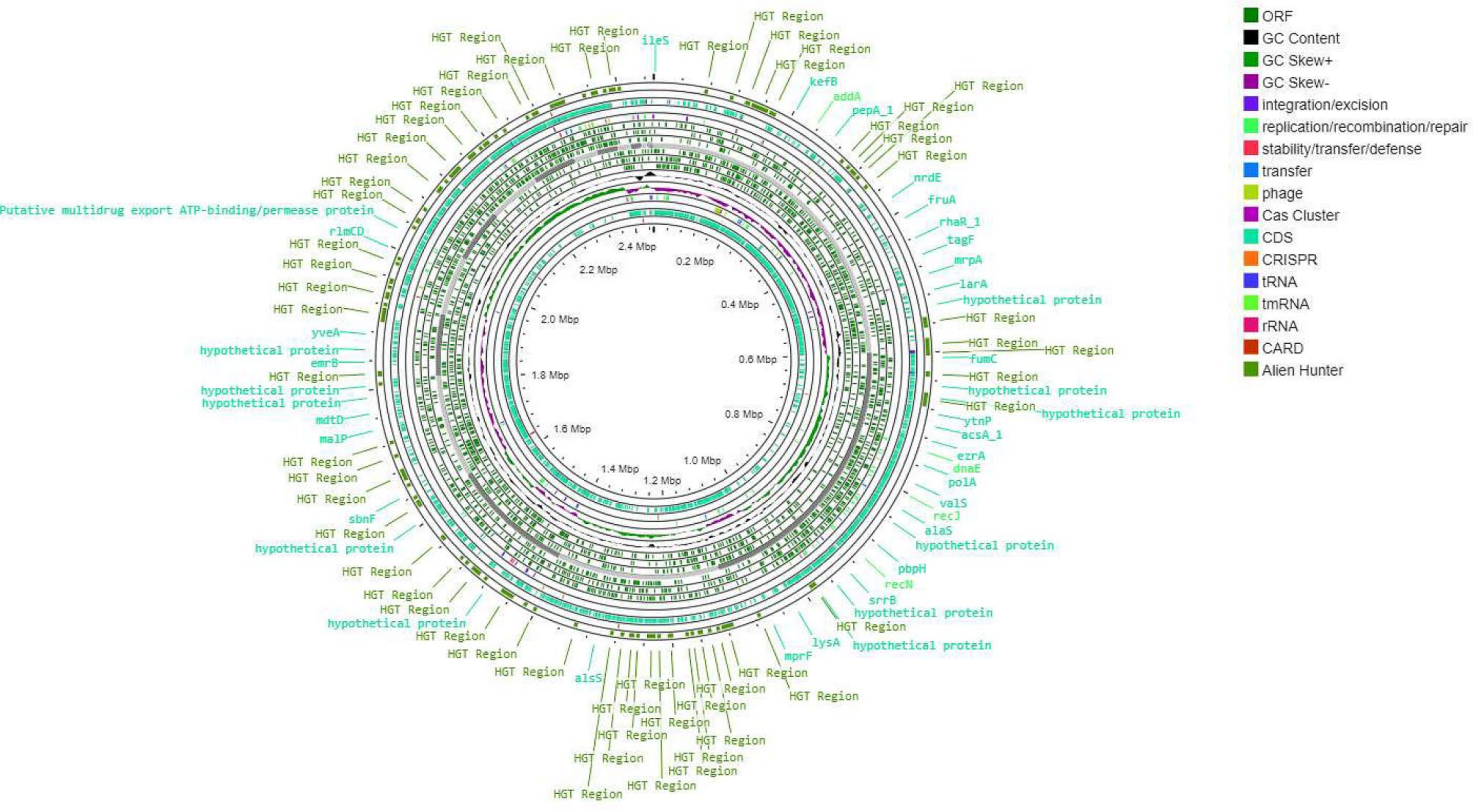
Circular genome map of NWU MKU1

**Fig. 4:**
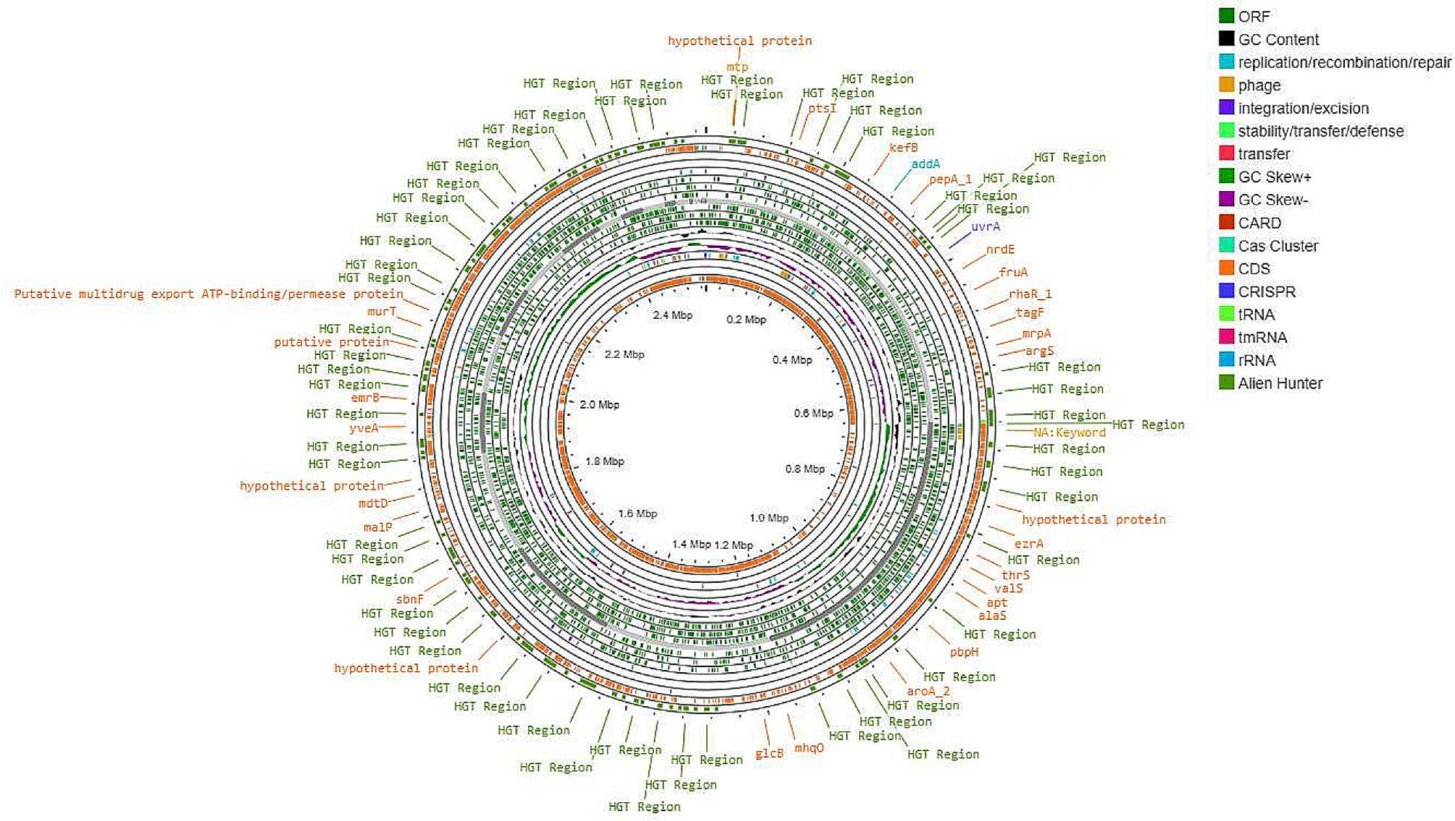
Circular genome map of NWU MKU2

**Fig. 5:**
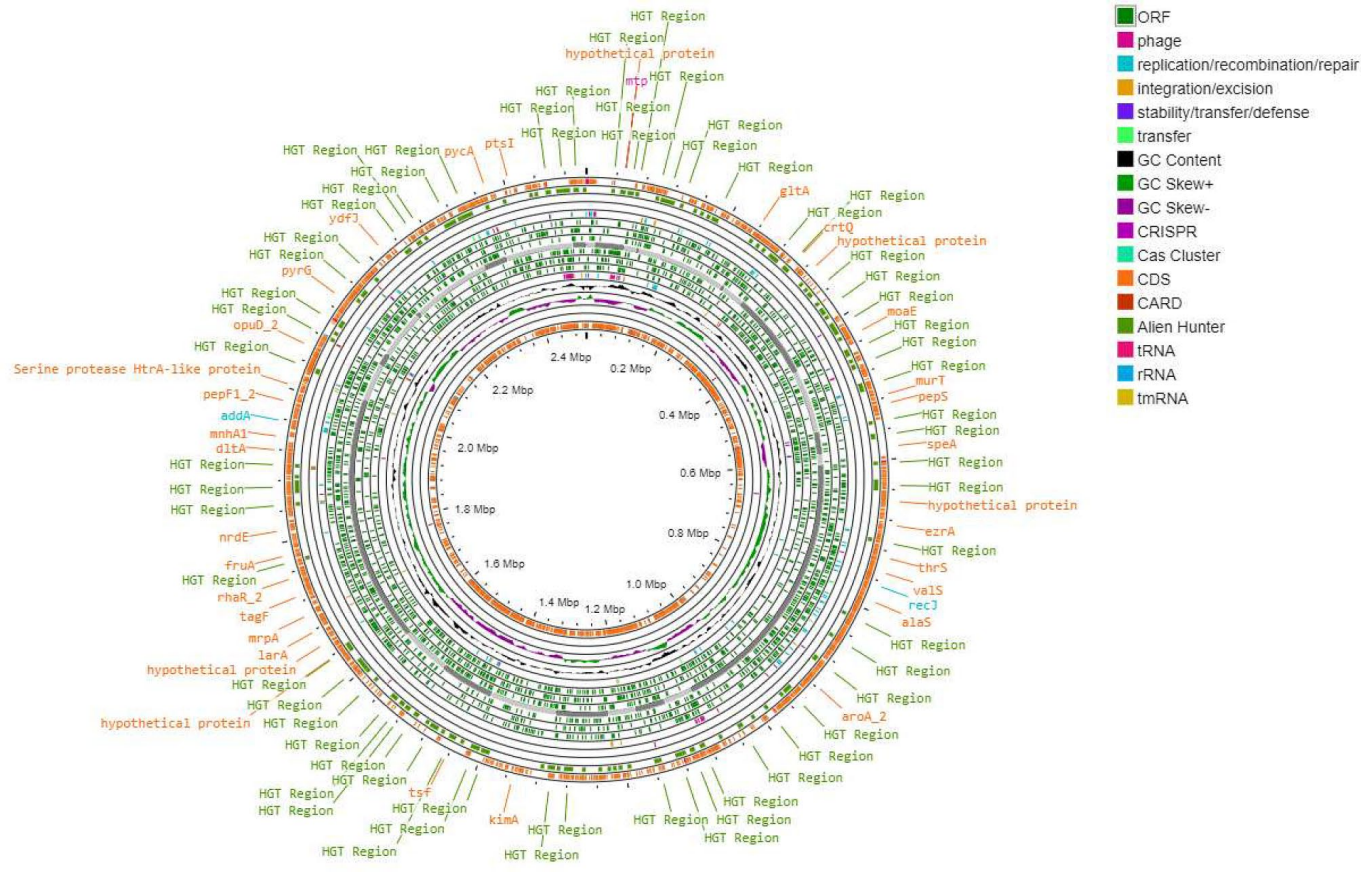
Circular genome map of NWU MKS3

Figures 3, 4 and 5 present the circular genome, illustrating several components, such as open reading frames (ORFs), GC content and GC skew, transfer RNA (tRNA), transfer messenger RNA (tmRNA), ribosomal RNA (rRNA), resistance genes, and clustered regularly interspaced short palindromic repeats (CRISPR) arrays, as well as their associated CRISPR-associated sequence (Cas) proteins, mobile genetic elements, and potential horizontal gene transfer events.

**Fig. 6:**
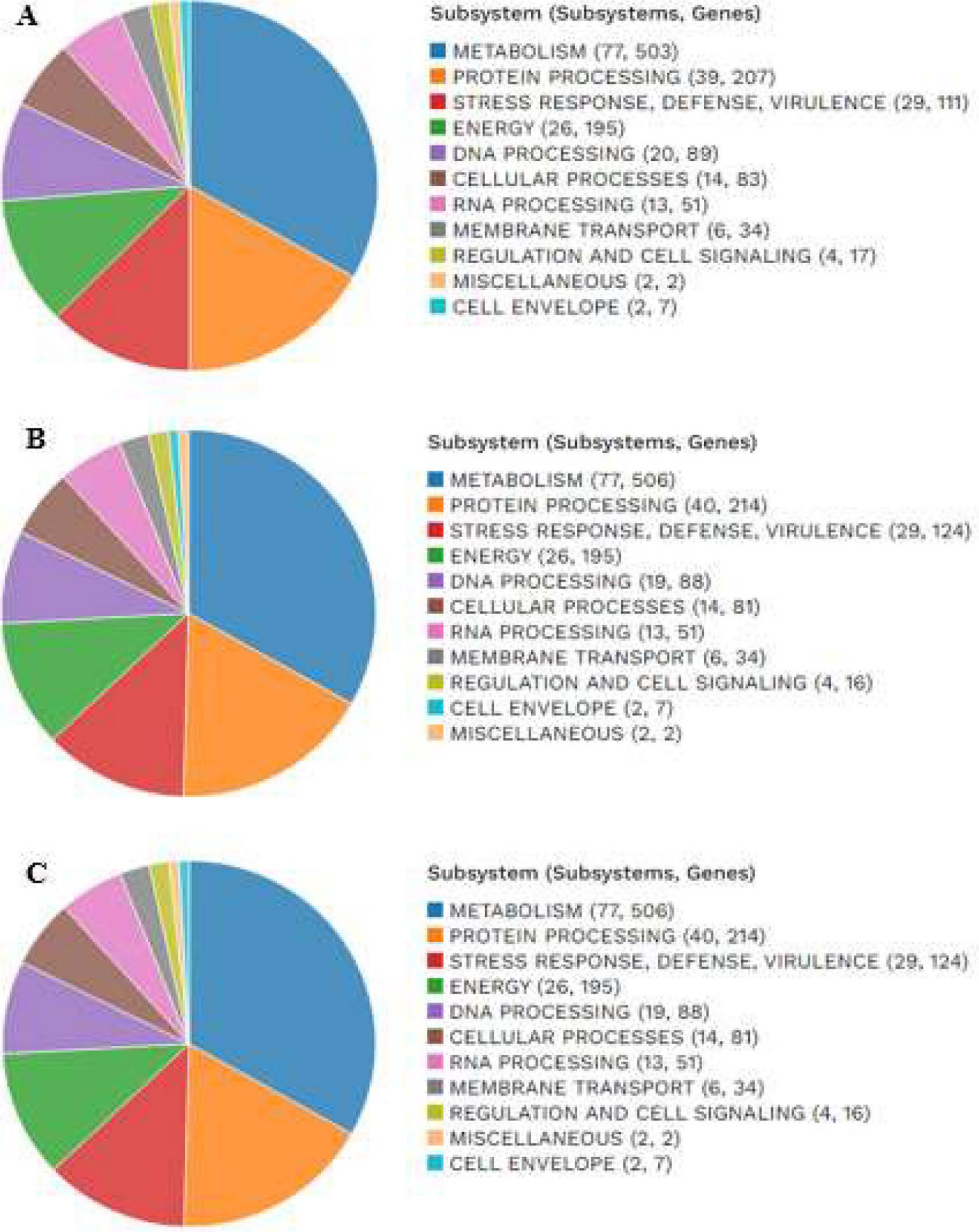
The subsystems and genes present in each genome: **A** – NWU MKU1, **B** – NWMKU2, **C** - NWU MKS3

The PATRIC annotation incorporates an examination of the subsystems that are distinctive to each genome. Figure 6 presents a comprehensive depiction of the subsystems pertaining to the genomes under investigation.

#### Antibiotic resistant, mobile genetic elements and virulence

Table 2 presents a summary of the AMR genes that have been identified in the genomesof the three isolates, along with their corresponding drug class and the resistance mechanisms associated with them. The findings derived from the online antibiotics RGI database revealed the presence of multiple antibiotic resistance genes (ARGs) within the isolates’genomes. Various ARGs found in the genomes encompass *tet* (A/B/T/O), *mac* B, *evg* A, *mar* A, *mef* B, *lmr* S, *bcr* A, *pat* B, NmcR, *optr* A, *erm* K, aac(6´), *bla* Z, and *dfr* C. Thesegenes encode resistance against various drug classes including aminoglycoside, cephalosporin, peptide, macrolide, fluoroquinolone, glycopeptide, penem, lincosamide, tetracycline, and phenicol, among others. The genomes of the isolates exhibit various mechanisms of AMR such as drug inactivation, alteration of antibiotic targets, antibiotic efflux, reduced permeability to antibiotics, and protection of antibiotic targets.

The genes that are potentially responsible for key virulence factors, including toxin (cytolysin), adherence (autolysin, elastin binding protein, and fibrinogen binding protein), immunological evasion (capsule and polyglutamic capsule), and enzymes (lipase and nuclease), were identified as being present in all genomes (Table 3).

**Table 3:** Antibiotic and virulence determinants in the test genomes

Virulence factor	Related gene		
	Isolate NWU MKU1 (Staphylococcus haemolyticus)	Isolate NWU MKU2 (Staphylococcus haemolyticus)	Isolate NWU MKS3 (Staphylococcus haemolyticus)
**Adherence**			
Autolysin	*atl*	*atl*	*atl*
Elastin binding-Protein	*ebp*	*ebp*	*ebp*
ser-Asp fibrinogen binding protein	*sdr*D	*sdr*D	-
**ser-Asp fibrinogen binding protein**	*sdr*E	*sdr*E	-
**Enzyme**			
**Lipase**	*lip*	*lip*	*lip*
Thermonuclease	*nuc*	*nuc*	*nuc*
**Immune evasion**			
**Capsule**	*cap*_*Sh*_	cap_Sh_	-
Polyglutamic Acid capsule	*cap*B	*cap*B	-
**Toxin**			
Cytolysin	*cylR2*	*cylR*2	-
**Antiphagocytosis**			
Capsule	*uge*	-	-
**Isolate**	**Drug Class**	**Antibiotic Resistant Gene (ARG)**	**Resistance Mechanism**
**NWU MKU1 (** ***Staphylococcus haemolyticus*** **)**	Fluoroquinolone, glycopeptide, macrolide, peptide, tetracycline, cephalosporin, monobactam, carbapenem	*sep*A, *sdr*M, *nor*(C/B), *van*(K, R,H, T/Y), PC1 beta-lactamase (*bla*Z), *dfr*C, *mec*D, *tet*(A/B/T/O), *mac*B, *evg*A, *mar*A, *mef*B, LmrS, *bcr*A, *pat*B, NmcR, *optr*A, PJM-1, *fex*A, *lmr*B, cmx, *sal*C, *cfr*A, *erm*K, *aac*(6´)	Antibiotic efflux, antibiotic inactivation, antibiotic target alteration, reduced permeability to antibiotic
**NWU MKU2 (** ***Staphylococcus haemolyticus*** **)**	Fluoroquinolone, glycopeptide, tetracycline, aminoglycoside, penam, peptide, cephalosporin, macrolide, aminocoumarin, carbapenem, monobactam, diaminopyrimidine, lincosamide, rifamycin, mupirocin-like antibiotic, streptogramin antibiotic, phenicol	*sep*A, *sdr*M, *nor*C, *van*(Y/T/S/H/R/L/O/G/R/B), *mgr*A, *msb*A, NmcR, EstT, *tet*(T/A/O/W/B), PmrF, *nor*A, *bcr*A, *alm*E, sta, RanA, *nov*A, *bac*A, *mac*B, BEL-2, *bae*S, *arl*(R/S), *dfr*C, LpsB, adeL, *ole*C, *leu*O, PJM-1, cmlV, *optr*A, *erm*K, *fex*A, EC-18, MexS, *aac*(6´), *erm*K, *fex*A, EC-18, MexS	Antibiotic efflux, antibiotic target alteration, antibiotic inactivation, antibiotic target protection, antibiotic target replacement, reduced permeability to antibiotic
**NWU MKS3 (** ***Staphylococcus haemolyticus*** **)**	Fluoroquinolone, glycopeptide, macrolide, aminoglycoside, phenicol, beta-lactam, tetracycline, sulfonamide, fluoroquinolone, trimethoprim resistant-dihydrofolate reductase	*gyr*A, *sep*A, *sdr*M, LmrS, *mgr*A, *optr*A, *emr*(K/B), *mep*A, cmx, *dfr*C, CAR-1, *sal*C, *sul*4, *ram*A, CMY-89, CRP, *aac*(6´), arlR, NmcA beta lactamase, *tet*(A/B/O/W), *nor*(C/B), *van*(M/Y/P/T/S/K/H/O/T), *sat*A	Antibiotic efflux, antibiotic inactivation, antibiotic target alteration, reduced permeability to antibiotic, antibiotic target protection

The prophage search conducted using PHASTER revealed the sequences coding for presence of one intact prophage for NWU MKU1 and three intact prophages for NWU MKU2. No questionable or incomplete prophages were detected for these two genome samples. Five prophage sequences were identified in NWU MKS3 genome, consisting of two intact prophages and three incomplete prophages. The MGEs identified using the PATRIC database for each genome encompass various functional categories, namely, transfer, phage, replication/recombination/repair, integration/excision, and stability/transfer/defense. Based on the results obtained from the MobileElementFinder, it was observed that NWU MKU1 genome contained two insertion sequences (ISs) belonging to families IS3 and IS6. Both NWU MKU2 and NWU MKS3 genomes each contain a single IS element belonging to the IS6 family.

#### Phylogeny

**Fig. 7:**
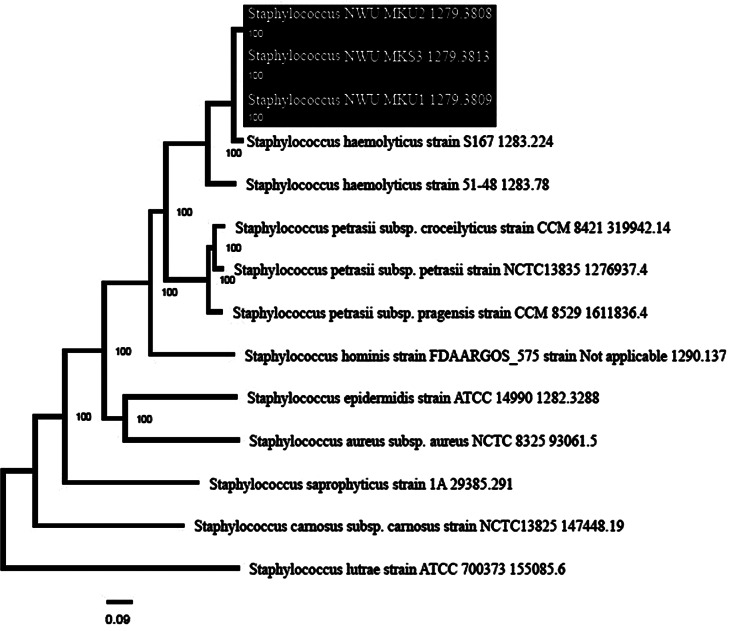
The phylogenetic tree illustrates the evolutionary relatedness between the genomes of the test isolates (NWU MKU1, NWU MKU2 and NWU MKS3) and a subset of *Staphylococcus* strains. The phylogenetic tree was constructed using PATRIC Bacterial Genome Tree platform. Highlighted strains are the test strains

The phylogenetic tree shows that the test isolates (NWU MKU1, NWU MKU2 and NWU MKS3) belong to the same clade. In addition, all test isolate genomes were observed to have the most common ancestral lineage with *S. haemolyticus* strain S167 (Fig. 7).

## Discussion

Although the use of the cultural-base technique is essential for the identification, enumeration, and detection of the viability, virulence, and AMR phenotypic profile of pathogenic bacteria, the extensive and laborious process of culturing, together with the inability to culture certain types of bacteria, present a significant limitation of this technique [60]. Hence, the adoption of WGS in this study to fully characterise the bacteria isolates.

*S. haemolyticus* has been previously documented as a one of the neglected foodborne pathogens with high AMR profile [61, 62]. The strain is recognized as an emerging opportunistic pathogen, and its association with other bacteria has led to the transfer of ARGs, thus making it a potential threat. This study confirmed the presence of multiple resistance genes in *S. haemolyticus*, as several antibiotic resistance genes (ARGs), including *sep* A, *nor* C, PmrF, *dfr* C, *optr* A, *erm* K, and *aac*(6´), were detected in the genomes of the isolates. These genes confer resistance to several antibiotic groups, including beta lactams, aminoglycosides, linezolids, tetracyclines, and macrolides. Along with the ARGs detected, resistance mechanisms, including efflux, antibiotic target al.teration and inactivation, and reduced permeability to antibiotics, were identified. The antibiotic resistance genes identified in the isolates under investigation corroborate the isolates’ phenotypic resistance profiles. The phenomenon of heightened antibiotic resistance exhibited by *Staphylococcus* species has been previously reported in food-producing animals and their products [63, 64] which aligns with the current findings. This can be attributed to the escalated use of these antibiotics in the management of staphylococcal infections.

The lack of amplification of the biofilm determinants *ica* A and *ica* D is typical of NAS species. This finding is in agreement with the findings of Andrade et al. [65] who argued that the majority of staphylococci isolated in their study could generate biofilms. The authors further added that the presence of the *bap* gene was exclusively observed in coagulase-negative staphylococci (CNS), whereas the *ica* operon genes were predominantly discovered in *S. aureus* isolates, indicating that the CNS primarily forms biofilms through the action of the *bap* gene. Based on the assertion of Pain et al. [66] the identification of biofilm-producing *S. haemolyticus* isolates that demonstrate resistance to oxacillin (*mec* A) and aminoglycosides (*aac* A-*aph* D) implies a greater probability of invasion, and the absence of these traits unequivocally indicates a commensal strain. Based on the results of this investigation, it was inferred that the isolates studied exhibit invasive characteristics because they harbor genes that confer resistance to amoxicillin, a penicillin antibiotic. The results also indicated that the presence of the *aac* (6´) gene is associated with resistance to aminoglycosides. Hence, it could be deduced that the MDR characteristics of the isolates are influenced by their ability to form biofilms, as biofilms serve to shield pathogens from the detrimental impacts of antibiotics. However, it is noteworthy to state that while *ica* A and *ica* D did not amplify in the test *S*. *haemolyticus* strains, the *bap* gene was not precisely screened. Consequently, the isolation of *Staphylococcus* strains from milking equipment, food sources, water, food processing surfaces, and machinery has become a challenging task.

The isolates demonstrated a significant occurrence of ARGs among their collection of accessory genes. This result was expected due to the frequent association of infections caused by MDR bacteria with *S. haemolyticus.* A similar argument was presented by Chang et al. [67], who stated that the exchange of genetic materials that occurs during interactions between microbial populations frequently results in the emergence of resistant strains. The transmission of genetic material may occur through horizontal gene transfer (HGT). Resistance determinants may be transmitted from bacterial chromosomes to mobile genetic elements (MGEs) during this process. The sample genomes contained numerous MGEs, including insertion sequences (ISs) and prophages, according to their genomic compositions. To adapt, these bacteria have obtained advantageous core mutations or MGEs. Multiple authors have documented the transfer of genetic material from bacterial chromosomes to MGEs, including plasmids, prophages, insertion sequences, and transposons [54, 68].

The analysis of PHASTER annotations revealed the significant presence of intact prophage-associated genes in NWU MKU2 and NWU MKS3, suggesting that many bacteriophage remnants had infiltrated these particular strains. The susceptibility of the aforementioned strains to infection may have been facilitated by their deficiency in the “immune system” against bacteriophages and ISs. In contrast, the NWU MKU1 strain exhibited a reduced number of prophages and coding sequences associated with IS elements. It is possible that the presence of the CRISPR‒Cas system plays a protective role in preventing invasion by bacteriophages. This argument stems from the conclusions of Alkhnbashi et al. [69] who reported that the utilisation of CRISPR in conjunction with related Cas genes serves as an adaptive immune system response to mobile genetic components, particularly bacteriophages.

Several virulence factors were found to be shared among all the isolates. One illustrative example of such a factor is the type II secretion system, which is responsible for the secretion of properly folded proteins, including lipase (*lip* A). Other factors include adherence factors, such as autolysin (*atl*), elastin binding protein (*ebp*) and fibrinogen binding protein (*sdrE*); immune evasion factor (*cap* B); and cytolysin (*cyl* R2) toxin. Typically, elastin-binding protein and fibrinogen-binding protein facilitate bacterial cell binding and attachment to host cells, while cytolysins play a critical role in determining the virulence of *S. haemolyticus*. These attributes contributed to the invasive and virulent tendencies of the tested isolates. These findings are consistent with the findings of Czekaj et al. and Eltwisy et al. [70, 71] who reported that processes of bacterial adhesion and internalisation are facilitated by the presence of biofilms and fibronectin-binding proteins (*fnbp*), and upon entry of *S. haemolyticus* into host cells, the organism releases toxins and enzymes that facilitate tissue damage, induce the activation of proinflammatory cytokines, and trigger the death of host cells. In addition, the presence of an antiphagocytosis gene (*uge*) was detected; however, this gene was found only in the genome of NWU MKU1. Hence, the pathogenic characteristics that were identified in all the strains are exemplified by their virulence.

Upon conducting a phylogenetic analysis of the test strains in relation to other strains, it was ascertained that the strains in question exhibited a strong affinity for the Korean strain (*S. haemolyticus* S167). This phenomenon may be ascribed to the outcome of population dynamics. Indeed, the past thirty years have been marked by heightened economic interaction between Africa and Asia, which has resulted in an expansion of international travel between the two continents [72]. South Africa and the Republic of Korea (ROK) maintain amicable economic relations owing to their complementary economies. The demand for consumer products in South Africa and the demand for raw materials in Korea are the driving forces behind this relationship. The ROK’s most significant trading partner in Africa is South Africa. Population migration can result in the transport of pathogens and the potential for genetic material to be exchanged. Owing to the heightened economic migration and social interaction between the two regions, our isolates are phylogenetically similar to strains found in Korea.

Finally, it is noteworthy to state that a major limitation of this study is the number of samples sequenced. As it was earlier stated, 5 MDR isolates were identified, however, only 3 were sequenced.

## Conclusion

The purpose of this research was to characterise three MDR and biofilm-forming strains of *S. haemolyticus* that were isolated from bovine milk. A better understanding of the complete DNA sequences of the NWU MKU1, NWU MKU2, and NWU MKS3 strains was achieved through the utilisation of the WGS method. The profusion of virulence and resistance genes identified within the genomes of these isolates and theircapacity to form biofilms serve as indicators of their potential public implications, the gravity of the infections they can induce in both animals and humans, and the challenges associated with their control. The heterogeneous MGEs detected in the test strains indicate the dynamic nature of each strain throughout its evolutionary lineage. The escalating prevalence of MDR *S. haemolyticus* in the milking parlour and the broader food chain has the potential to cause severe health concerns for animals, the environment, and humans. Consequently, it is crucial to implement adequate surveillance and monitoring systems, as well as effective farm management and adherence to good manufacturing practices, to ensure the safety and sustainability of food production. Furthermore, alternative treatment options are recommended for treating AMR.

## Data Availability

The datasets generated and/or analysed during the current study are available with the following accession numbers: JAVSMG000000000, JAVSMH000000000, and JAVSMI000000000.
